# Intracellular behavior of *Nocardia seriolae* and its apoptotic effect on RAW264.7 macrophages

**DOI:** 10.3389/fcimb.2023.1138422

**Published:** 2023-02-28

**Authors:** Wenwen Liu, Yuting Deng, Aiping Tan, Fei Zhao, Ouqing Chang, Fang Wang, Yingtiao Lai, Zhibin Huang

**Affiliations:** ^1^ Key Laboratory of Fishery Drug Development of Ministry of Agriculture and Rural Affairs, Pearl River Fisheries Research Institute, Chinese Academy of Fishery Sciences, Guangzhou, China; ^2^ Guangdong Provincial Key Laboratory of Aquatic Animal Immunology and Sustainable Aquaculture, Guangzhou, China; ^3^ College of Fisheries and Life Science, Shanghai Ocean University, Shanghai, China; ^4^ Key Laboratory of Control of Quality and Safety for Aquatic Products of Ministry of Agriculture and Rural Affairs, Chinese Academy of Fishery Sciences, Beijing, China

**Keywords:** *Nocardia seriolae*, macrophages, intracellular bacteria, apoptosis, survival mechanism

## Abstract

*Nocardia seriolae*, an intracellular gram-positive pathogen, is prone to infecting immunocompromised and surface-damaged fish, causing serious losses to the aquaculture industry. Although a previous study has demonstrated that *N. seriolae* infects macrophages, the persistence of this bacterium in macrophages has not been well characterized. To address this gap, we used the macrophage cell line RAW264.7, to investigate the interactions between *N. seriolae* and macrophages and deciphered the intracellular survival mechanism of *N. seriolae.* Confocal and light microscopy revealed that *N. seriolae* entered macrophages 2 hours post-inoculation (hpi), were phagocytosed by macrophages at 4–8 hpi, and induced the formation of multinucleated macrophages by severe fusion at 12 hpi. Flow cytometry, evaluation of mitochondrial membrane potential, release of lactate dehydrogenase, and observation of the ultrastructure of macrophages revealed that apoptosis was induced in the early infection stage and inhibited in the middle and later periods of infection. Additionally, the expression of Bcl-2, Bax, Cyto-C, Caspase-3, Capase-8, and Caspase-9 was induced at 4 hpi, and then decreased at 6–8 hpi, illustrating that *N. seriolae* infection induces the activation of extrinsic and intrinsic apoptotic pathways in macrophages, followed by the inhibition of apoptosis to survive inside the cells. Furthermore, *N. seriolae* inhibits the production of reactive oxygen species and releases large amounts of nitric oxide, which persists in macrophages during infection. The present study provides the first comprehensive insight into the intracellular behavior of *N. seriolae* and its apoptotic effect on macrophages and may be important for understanding the pathogenicity of fish nocardiosis.

## Introduction

*Nocardia seriolae* is a branching filamentous rod-shaped intracellular gram-positive pathogen belonging to the order *Actinomycetales*, family *Nocardiaceae*, and genus *Nocardia* (Liu et al., 2023). It mainly causes chronic infection in fish, with a long incubation period and a high mortality rate of up to 100%, which causes large economic losses to the aquaculture industry. Fish nocardiosis, caused by *N. seriolae*, is a systemic chronic granulomatous disease that has been reported in 42 freshwater and saltwater species, and more frequently in *Micropterus salmoides*, *Channa argus*, *Trachinotus ovatus*, and *Lateolabrax japonicus* in China (Liu et al., 2023). The typical symptoms of nocardiosis include white nodules in the internal organs and well-defined granulomas of histopathological structures. White granulomas are products of interactions between *N. seriolae* and the immune system of fish, and are composed of *N. seriolae-*infected macrophages and epithelioid cells (Liu et al., 2023). In a previous study, we found that *N. seriolae* was phagocytized by macrophages in an experimentally infected transparent tiger spiny fish (*Puntius tetrazona*), resulting in systemic nocardiosis (Wang et al., 2017). Another study reported that *Nocardia brasiliensis* infection may induce macrophages and dendritic cells to differentiate into foamy cells (Meester et al., 2014). These results imply that macrophages play an important role in the defense mechanisms against invasion *by Nocardia* spp. Although several studies have reported nocardiosis in different fish species, the pathogenic mechanism of *N. seriolae* infections remains unclear.

With recent advances in cell biology and immunology, studies on the mechanisms of interactions between intracellular bacteria and their target cells have received increasing attention (Zhao et al., 2011). Macrophages, which are extremely important phagocytes in the immune system, can effectively phagocytose foreign antigens (e.g., pathogenic microorganisms and insoluble particles) and present antigenic peptides to CD4^+^ T cells to initiate specific immune responses (Nenan et al., 2005). Pathogen–cell interactions can induce cell death *via* three modes – apoptosis, necrosis, and pyroptosis – based on various factors, such as the type of pathogen, dose of infection, and infected target. The same pathogen can induce different modes of cell death in different cell types owing to their different immune responses (Lamkanfi and Dixit, 2010). In previous studies, *Shigella flexneri* inhibited epithelial cell necrosis, but triggered macrophage apoptosis. (Clark and Maurelli, 2007; Suzuki et al., 2005). Some intracellular bacteria, such as *Mycobacterium* spp., *Brucella* spp., and *Nocardia* spp., can invade the body *via* different routes, including the respiratory tract, digestive tract, skin, and mucous membrane. Upon interaction with macrophages, intracellular pathogens can maintain their own survival and reproduction by inhibiting oxidative cell death and apoptosis, and avoiding autophagy from the initial immune mechanism (Qin et al., 2017). Macrophages are the main target cells of *N. seriolae* for its survival and reproduction. However, the molecular mechanisms by which they adhere to, invade, and interact with macrophages have not yet been reported. To address this gap, it is crucial to use host macrophages to study the pathogenic mechanisms of *N. seriolae* infections. However, there are currently no commercialized macrophage cell lines from aquatic animals, and the use of primary macrophages obtained from fish tissues is associated with several potential issues, such as difficulty in *in vitro* passage, instability, and heterogeneity. In contrast to fish cells, the murine macrophage cell line RAW264.7, which has a rapid growth rate, is easy to culture, phenotypically resembles primary macrophages, and has been widely used to study interactions between pathogens and macrophages (Utaisincharoen et al., 2000; Touret et al., 2005; Lahiri et al., 2008; Ishibe et al., 2009).

To gain further insight into the interactions between *N. seriolae* and macrophages in nocardiosis, we established a RAW264.7 cell model for *N. seriolae* infection *in vitro*. We examined the internalization, replication, and intracellular performance of *N. seriolae* in the macrophages. Furthermore, *N. seriolae* infection-induced regulation of extrinsic and intrinsic apoptosis, as well as its association with immune responses, was determined. The present study provides the first comprehensive insight into the intracellular behavior of *N. seriolae* in macrophages, and may be important for understanding the pathogenicity of fish nocardiosis.

## Materials and methods

### Bacterial culture

The *N. seriolae* strain NK201610020 (NK) was previously isolated from a diseased hybrid snakehead (*Channa maculata ♀* × C. argus ♂) in Guangdong Province, China and was preserved in our laboratory. The NK strain was transformed with the fluorescent plasmid pRUALPGEN, as previously described (Wang et al., 2017), and the transformant was named GFP-NK. NK and GFP-NK *N. seriolae* strains were routinely sub-cultured on brain heart infusion (BHI) agar (OKA, China) and BHI agar supplemented with 50 μg/mL kanamycin (NCM Biotech, China) at 28°C for 3–4 days.

### Cell culture

RAW264.7 cell line were purchased from Solar Bio Biologicals (Beijing, China) and were cultured in complete medium Dulbecco’s modified Eagle’s medium (DMEM)-high sugar (Gibco, USA) supplemented with 10% fetal bovine serum (FBS; Gibco, USA) and 1% penicillin-streptomycin (Beyotime Biotechnology, Shanghai, China) at 37°C and 5% CO_2_. The cells were cultured in T25 cell bottles at 80-90% adventitious rate for transmission and used for follow-up experiments.

### Invasion assay in RAW 264.7 cells

Invasion assays were performed as previously described (Qin et al., 2017). The cells were digested with trypsin (Gibco, USA), resuspended in complete medium, adjusted to 1 × 10^6^ cells/mL using a cell counter (Count Star, IM 1200, China), added to the cell culture plate, and incubated overnight until the cells completely adhered to the plate walls. The GFP-NK strain was suspended in DMEM-high sugar with 10% FBS and then adjusted to 1 × 10^8^ cells/mL using a turbidimeter (HACH, TL2300, China). The bacterial suspension was inoculated into each well of a culture plate, equivalent to a multiplicity of infection (MOI; bacteria added per cell) of 100. Cells co-cultured with bacteria were incubated for 2, 4, 6, 8, or 12 h at 37°C and 5% CO_2._

### Microscopy

Cell invasion was observed at each time point by using an inverted fluorescence microscope. Briefly, co-cultured cells were washed three times with phosphate-buffered saline (PBS) (Gibco, USA) containing 100 µg/mL gentamicin. The colocalization and morphology of the GFP-NK strain and macrophages were observedusing an inverted fluorescence microscope (ZEISS, Axiovert200M, Germany).

For confocal microscopy observation, an invasion assay was performed as described above, except that the cells were inoculated in wells with 14-mm glass coverslips. After co-culture at each time point, end-of-infection cells were triple-cleaned using PBS supplemented with gentamicin at a final concentration of 100 μg/mL and incubated with Lyso-Tracker Red working solution (Beyotime Biotechnology, Shanghai, China) for 30 min at 37°C and 5% CO_2_. Thereafter, the cells were washed thrice with PBS, fixed with 4% paraformaldehyde, and stained with 200 μL DAPI (Beyotime Biotechnology, Shanghai, China). The cells were washed with PBS and observed under a confocal microscope (ZEISS LSM 900, Germany).

Transmission electron microscopy (TEM) was used to examine phagocytosis and the ultrastructure of the macrophages. Invasion assay was performed as described above. After co-culture at each time point, the end-of-infection cells were triple-cleaned using PBS supplemented with a final concentration of 100 μg/mL gentamicin, digested with trypsin for 5–10 min, and collected by centrifugation at 300 × *g* for 10 min at 4°C. The precipitated cells were fixed with glutaraldehyde electron microscope fixative (ZEISS, LIBRA120, Germany), stored at 4°C, and sent to Wuhan Servicebio Technology Co., Ltd., for TEM analysis.

### Observation of intracellular *N. seriolae* using flow cytometry

The number of macrophages infected with the GFP-NK strain was quantified by fluorescence-activated cell sorting (FACS) using a flow cytometer (Thermo Fisher Scientific, Attune NxT, USA) as previously described (Greer et al., 2022). Invasion assay was performed as described above. After co-culture at each time point, the cells were washed, digested, and collected by centrifugation as described above. The precipitated cells were fixed with 100 μL 70% ethanol. Flow cytometry was used to separate the infected (GFP-NK) cells from the uninfected (GFP-negative) cells. For each sample, 1000 cells were counted. Flow cytometry data were analyzed using FlowJo software, version 7.6, to determine the percentage of GFP^+^ cell population and the mean fluorescence intensity of the total cell population.

### Quantification of reactive oxygen species and nitric oxide levels in macrophages

Quantification of ROS levels in the macrophages was performed as previously described (Zou et al., 2017). RAW 264.7 cells were incubated in 96-well plates at a density of 1 × 10^5^ cells/well. The cells were left uninfected or infected with *N. seriolae* at MOI of 10:1 and incubated for 2, 4, 6, 8, and 12 h at 37°C with 5% CO_2._ After co-culture at each time point, end-of-infection cells were triple-cleaned using PBS supplemented with a final concentration of 100 μg/mL gentamicin, and then incubated with the cell-permeant fluorogenic ROS probe DCFH-DA (Solarbio, Beijing, China) at a final concentration of 10 μmol/L for 20 min at 37°C. After incubation, the cells were triplicated in solvent-free medium and fluorescence was measured using a multifunctional enzyme-labeled instrument (Bio Tek, ELx800, USA) at excitation and emission wavelengths of 525 and 488 nm, respectively. Triplicate wells were used for all experiments and the mean ± S.D. was calculated.

NO levels were assayed by estimating NO^2-^ concentration using the Griess reaction, as described in a previous study (Wang et al., 2004). After co-culture at each time point, the cells were washed, digested, and collected by centrifugation as described above. The precipitated cells were lysed and the supernatants incubated with Griess reagent (Solarbio, Beijing, China) (1:1 v/v) for 10 min at room temperature in a 96-well culture plate. Thereafter, the absorbance was spectrophotometrically measured at 540 nm using a multifunctional enzyme-labeled instrument (Bio Tek, ELx800, USA), and the NO^2-^ concentration was determined using a standard curve of NaNO_2_ (expressed as µmol/mL). Triplicate wells were used for all experiments and the mean ± S.D. was calculated.

### Apoptotic effect of *N. seriolae* on macrophages

Macrophage apoptosis was analyzed using the Annexin V-mCherry dual-fluorescence assay. After co-culture at each time point, the cells were washed, digested, and collected by centrifugation as described above. The precipitated cells were labeled by adding 194 μL Annexin V-mCherry binding buffer, 5 μL Annexin V-mCherry, and 1 μL SYTOX Green, according to the apoptosis assay kit (Beyotime Biotechnology, Shanghai, China). The samples were gently mixed and incubated at room temperature in the dark for 15 min. A minimum of 10,000 cells within the gated region were analyzed by flow cytometry (Thermo Fisher Scientific, Attune NxT, USA). The results were expressed as the percentage of apoptotic cells among all cells.

The effect of apoptosis on the mitochondrial potential was estimated using a mitochondrial cell membrane potential assay kit (JC-1). (Beyotime Biotechnology, Shanghai, China). After co-culture at each time point, the cells were washed, digested, and collected by centrifugation as described above. The precipitated cells were labeled with fluorescent probes (JC-1 dye) and subsequently analyzed for apoptosis and necrosis using flow cytometry (Thermo Fisher Scientific, Attune NxT, USA).

The effect of apoptosis on DNA fragmentation was determined as previously described (Bai et al., 2008). After co-culture at each time point, the cells were washed, digested, and collected by centrifugation as described above. The precipitated cells were extracted using an apoptosis-DNA ladder extraction kit (Beyotime Biotechnology, Shanghai, China), followed by 1.5% agarose gel electrophoresis. The gel was examined and photographed using an ultraviolet (UV) gel documentation system (Thermo Fisher Scientific, PS0123, USA).

The apoptotic effect on cytotoxicity was evaluated by determining the levels of lactate dehydrogenase (LDH) in the cell supernatant using a commercial LDH cytotoxicity detection kit (Beyotime Biotechnology, Shanghai, China). RAW 264.7 cells were incubated in 96-well plates and infected with *N. seriolae*, as described above. After co-culture at each time point, the cell culture plate was centrifuged at 400 × g for 5 min in a plate centrifuge. (Eppendorf, 5418R, Germany). The supernatant was then incubated with the LDH assay working solution according to the manufacturer’s instructions. Absorbance was measured at 490 nm using an enzyme-labeled instrument (BioTek, ELx800, USA) and expressed as a percentage of cytotoxicity by calculating the average absorbance values of three replicates and subtracting the absorbance value obtained from the background control.

### Measurement of apoptosis-related cytokine and cellular inflammatory factor levels

After co-culture at each time point, the cells were washed, digested, and collected by centrifugation as described above. Total cellular RNA was extracted using an RNA kit (Tiangen Biotech, Beijing, China) and reverse-transcribed into cDNA using a PrimeScript RT Reagent Kit (TaKaRa, Dalian, China), according to the manufacturer’s instructions. All quantitative PCRs were performed using TB Green^®^ Premix Ex Taq™ (TaKaRa, Dalian, China), according to the manufacturer’s instructions, and qPCR assays were performed on a QuantStudio 6 Flex Real-Time Fluorescence PCR instrument (Applied Biosystems, USA) equipped with real-time PCR-based gene expression profiling. The expression of apoptosis-related cytokines (Bax, Bcl-2, Caspase-3, -8, and -9, and Cyto-C) and cellular inflammatory factors (IL-6, IL-1β, and TNF-α) was detected at the transcriptional level, and the relative expression of each mRNA was calculated by the 2^-ΔΔCT^ method using GAPDH as an internal reference. The annealing temperatures, specific primers, and expected amplicon sizes of target genes are listed in **Supplementary Table 1**.

### Statistical analysis

All experimental data were validated by three independent experiments, and the experimental results were processed using GraphPad Prism 7.0 (San Diego, CA, USA) and SPSS statistical software (version 25.0; IBM Corp., Armonk, NY, USA). One-way analysis of variance (ANOVA) was used for comparison between groups, with *P*<0.05 indicating statistically significant differences, and *P* < 0.01 indicating a highly significant difference.

## Results

### Intracellular behavior of *N. seriolae* in the macrophage cell line

**Figure 1:**
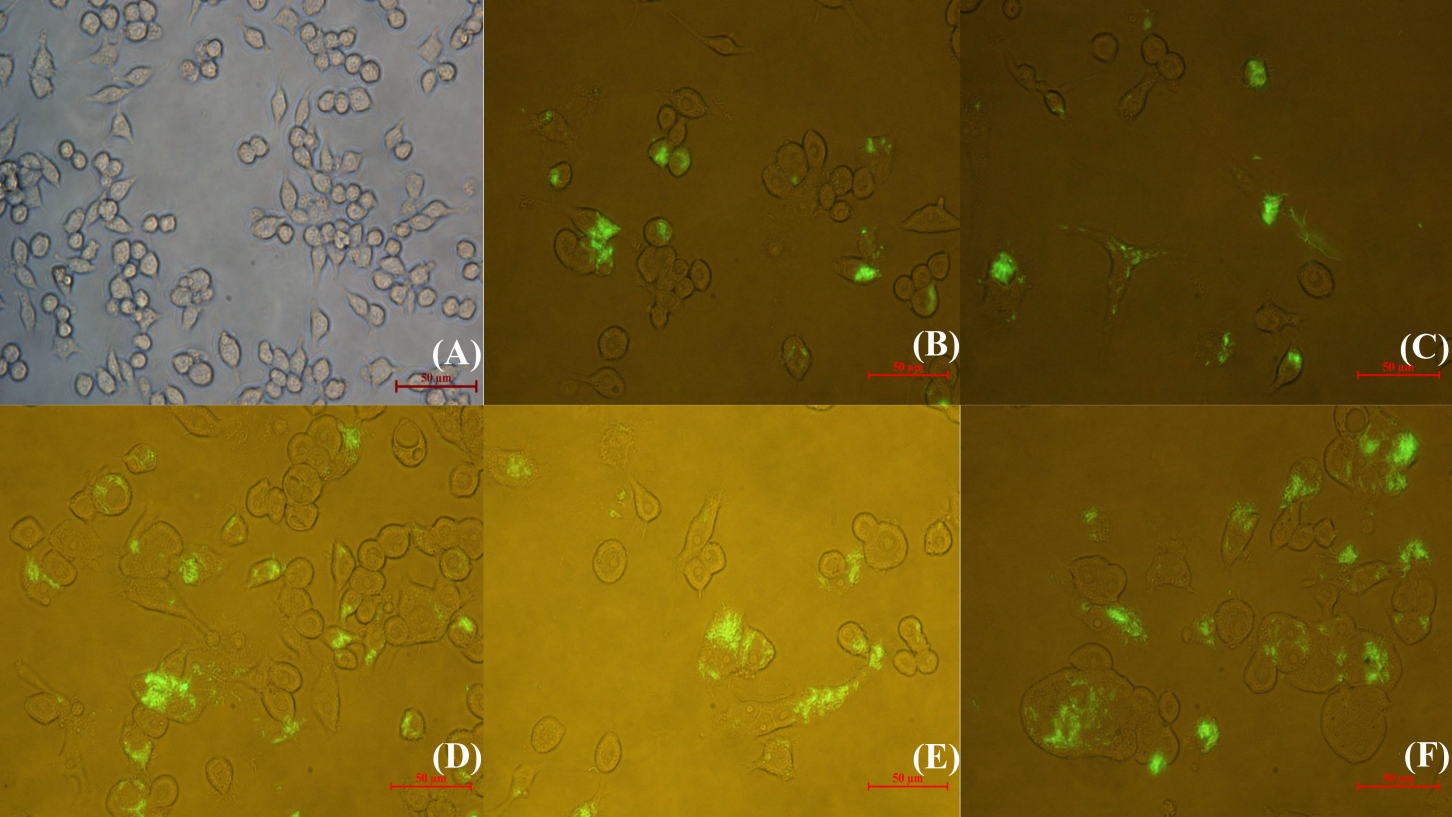
Inverted fluorescence microscopic observation of RAW264.7 infected with GFP-*N. seriolae.* **(A)** uninfected cells; **(B)** 2 hpi; **(C)** 4 hpi; **(D)** 6 hpi; **(E)** 8 hpi; **(F)** 12 hpi.

The intracellular behavior of *N. seriolae* was observed by inverted fluorescence microscopy at different time points after infection. Uninfected cells are shown in **Figure 1A**. After infection, a small quantity of *N. seriolae* entered the macrophages at 2 hours post-infection (hpi), at which time most macrophages did not show obvious changes in shape (**Figure 1B**). As the infection time increased from 4 to 8 hpi, the number of bacteria entering the cells gradually increased, and some macrophages began to show ruptured cell membranes, increased size, and significant deformation (**Figures 1C–E**). At 12 hpi, a large number of bacteria had filled the entire cytoplasm and proliferated. Furthermore, the macrophages fused severely and increased in size to form multinucleated macrophages (**Figure 1F**).

**Figure 2:**
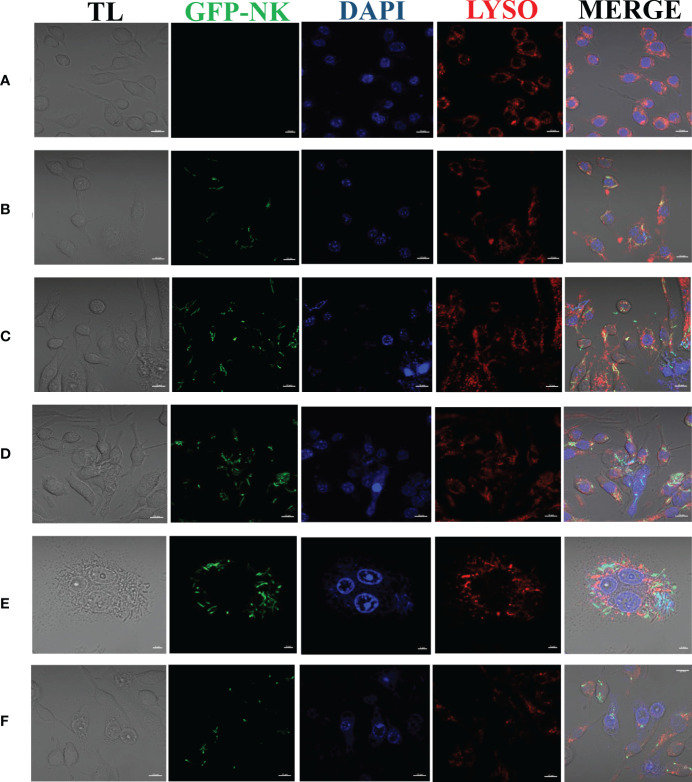
Laser confocalization of RAW264.7 infected with GFP-*N. seriolae.* **(A)** uninfected cells; **(B)** 2 hpi; **(C)** 4 hpi; **(D)** 6 hpi; **(E)** 8 hpi; **(F)** 12 hpi.

The colocalization of *N. seriolae* and macrophages was observed using confocal microscopy. The uninfected control cells were in good apposition and had a normal morphology; the nuclei were intact and evenly stained, and the lysosomes surrounded the cells diffusely (**Figure 2A**). At 2 hpi, a small number of cells were deformed; some bacteria entered the interior of the cells and combined with the lysosomes (**Figure 2B**). Cell fusion was observed at 4 hpi. Some cells showed cell membrane rupture and nuclear lysis, indicating apoptosis as determined by DAPI staining. Furthermore, the number of bacteria, in combination with intracellular lysosomes, increased significantly (**Figure 2C**). At 6 hpi, the bacteria combined with lysosomes and partially entered the nucleus (**Figure 2D**). At 8 hpi, the cell membrane was completely ruptured, the cells fused together in an irregular shape, the nucleoplasm solidified, lysosomal staining became lighter, and a large number of bacteria appeared to proliferate inside the cells (**Figure 2E**). At 12 hpi, many cells exhibited nuclear lysis or solidification, severe cell necrosis, and ruptured cell membranes, leading to the loss of lysosomes and other organelles. Some bacteria also adhered to the lysosomes or were phagocytosed by the remaining normal cells (**Figure 2F**).

### Ultrastructure and quantitative analysis of macrophages infected with *N. seriolae*

**Figure 3:**
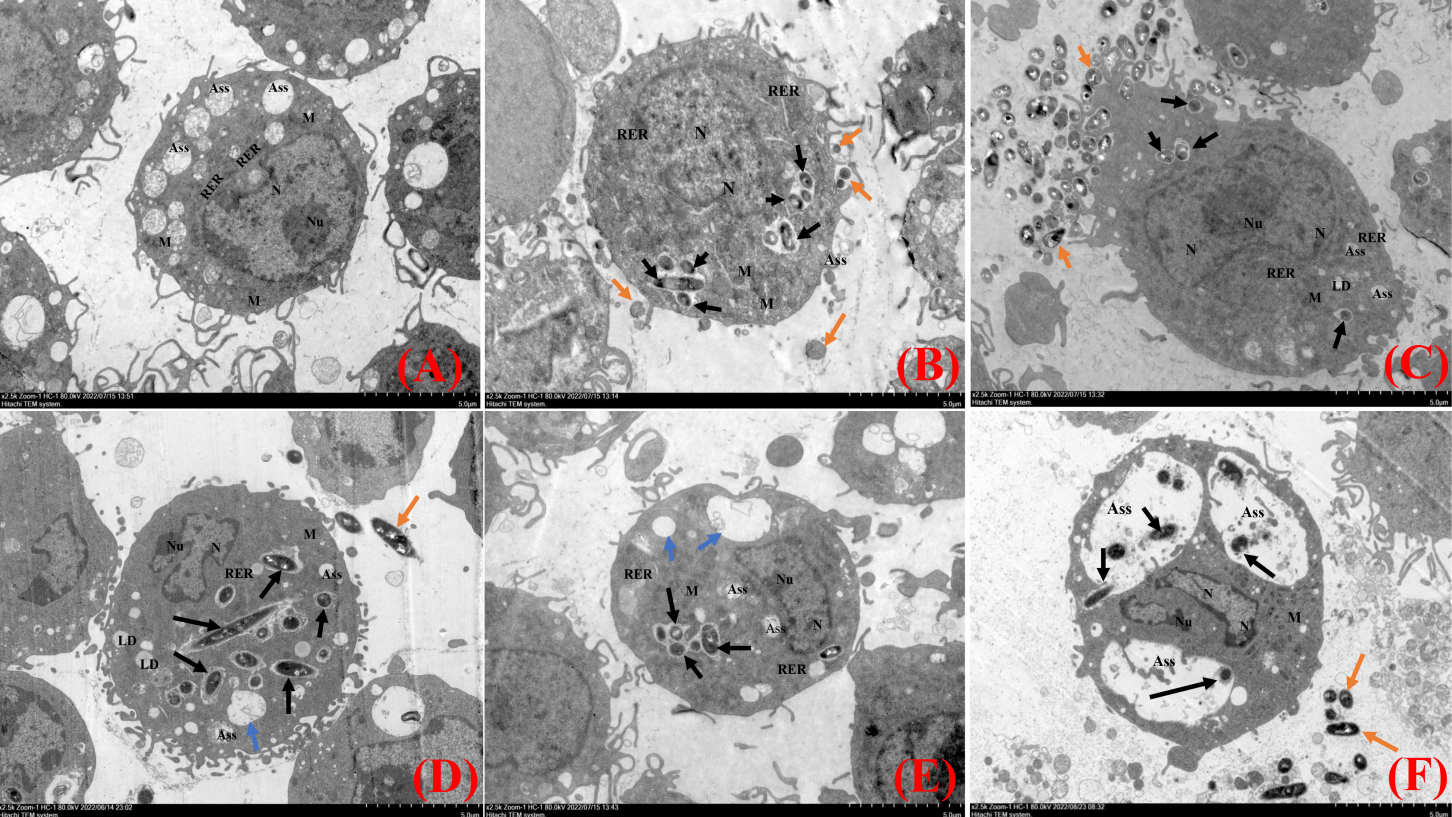
Transmission electron microscopy analysis of RAW264.7 infected with *N. seriolae.* **(A)** Uninfected cells, **(B)** 2 hpi, **(C)** 4 hpi, **(D)** 6 hpi, **(E)** 8 hpi, and **(F)** 12 hpi. Black arrows indicate intracellular bacteria, orange arrows indicate extracellular bacteria, and blue arrows indicate cell vacuoles.

TEM observations demonstrated that the uninfected cells were morphologically intact, with no damage to organelles, and showed a high number of intracellular phagocytosed lysosomes and an intact cell membrane with obvious pseudopods (**Figure 3A**). At 2 hpi, *N.seriolae* was intracellularly wrapped by membrane structures and a small number of extracellular bacteria adhered to the cell surface. The morphology of the cells did not change significantly and the nucleus was irregularly shaped with a blurred membrane, increased heterochromatin, and a large nucleolus. The structures of the mitochondria, endoplasmic reticulum, and other organelles showed no obvious changes compared to those of the uninfected cells (**Figure 3B**). At 4 hpi, the cells were irregularly shaped with swollen intracellular organelles. The mitochondria were partially swollen, with a pale local matrix and reduced cristae, and the rough endoplasmic reticulum was partially dilated and degranulated. A small number of bacteria were visible in the cells (**Figure 3C**). At 6 hpi, more autophagic lysosomes were present intracellularly and vacuoles were observed. Some bacteria were widely and freely distributed throughout the cytoplasm (**Figure 3D**). At 8 hpi, the cells shrank and became rounded, and the number of pseudopods reduced. Many bacteria were visible intracellularly and the number of vacuoles increased significantly (**Figure 3E**). At 12 hpi, the nucleus was irregularly shaped with a large rupture of the nuclear membrane and large nuclear vesicles. More bacteria were visible inside the cells, with significantly enlarged autophagic lysosomes, inhibition of lysosomal fusion, and severe damage to the internal cell structure (**Figure 3F**).

**Figure 4:**
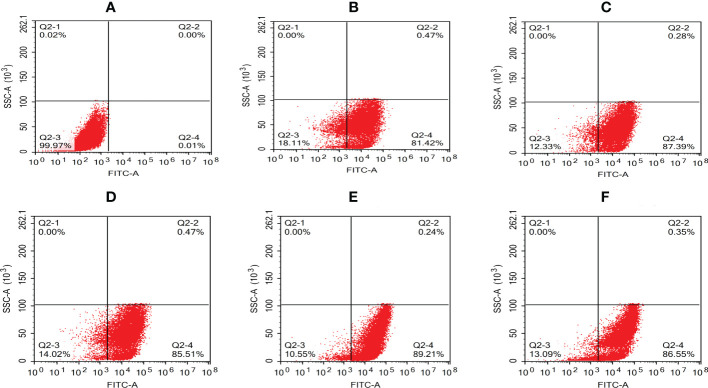
Quantative analysis of macrophages infected with *N. seriolae* by flow cytometry. **(A)** Uninfected cells: **(B)** 2 hpi, **(C)** 4 hpi, **(D)** 6 hpi, **(E)** 8 hpi, and **(F)** 12 hpi.

**Figure 5:**
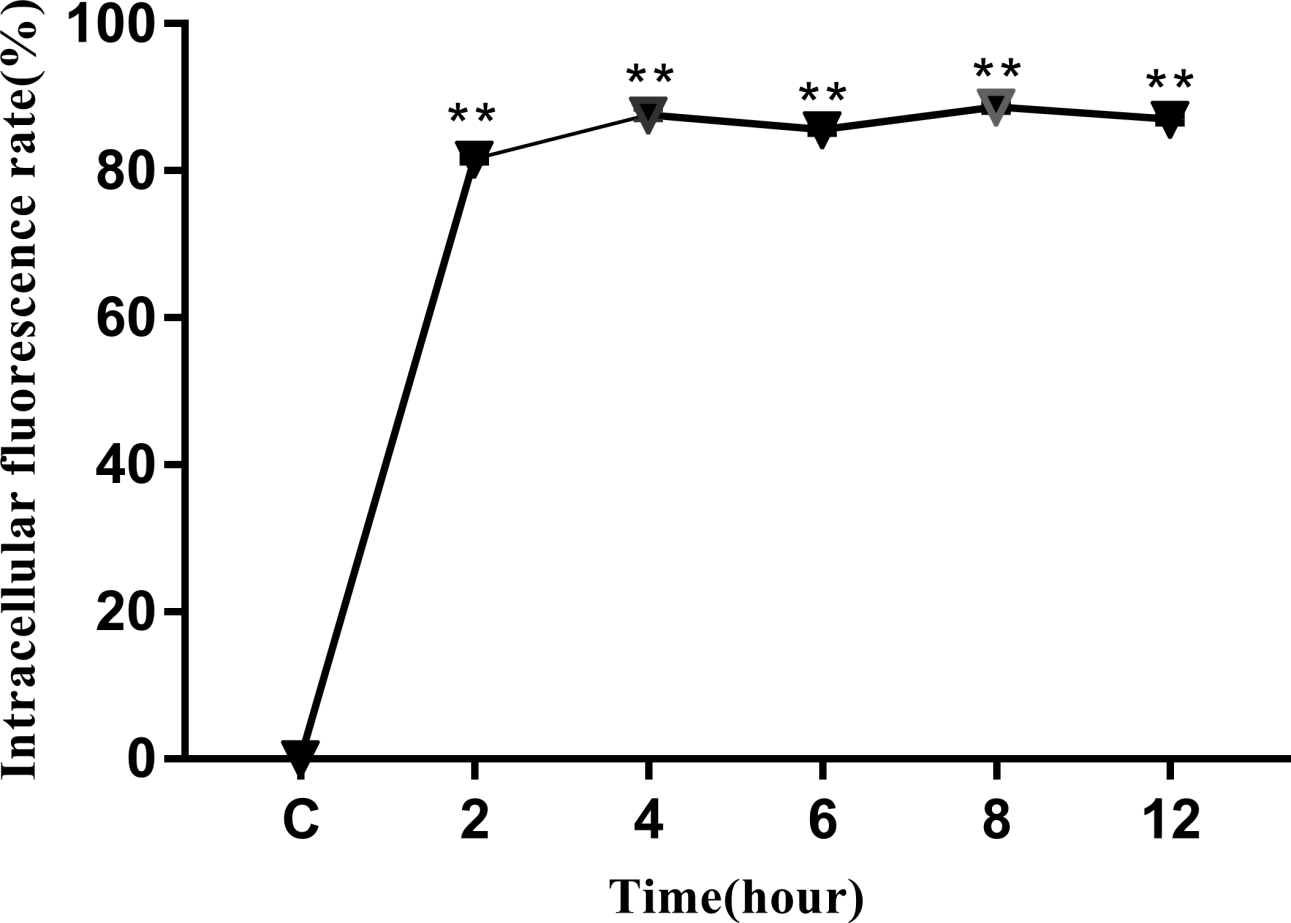
Intracellular fluorescence rate of RAW264.7 infected with *N. seriolae.* Data are presented as mean ± SD of three independent experiments. **P < 0.01 as compared with the control group.

The intracellular behavior of *N. seriolae* in the macrophage cell line was quantified by flow cytometry. A significant difference in the infected cell population was observed between the time points after infection compared with that in the control group (uninfected cells; **Figure 4A**). The percentage of cells showing fluorescence increased from 81.42% at 2 hpi to 87.39% at 4 hpi, decreased slightly at 6 hpi, peaked at 8 hpi, reached 89.21%, and then decreased to 86.55% at 12 hpi (**Figures 4B–F**). The results of the statistical analysis showed highly significant differences (*P* < 0.01) between each experimental group and the control group (**Figure 5**).

### Quantification of ROS and NO levels in macrophages

**Figure 6:**
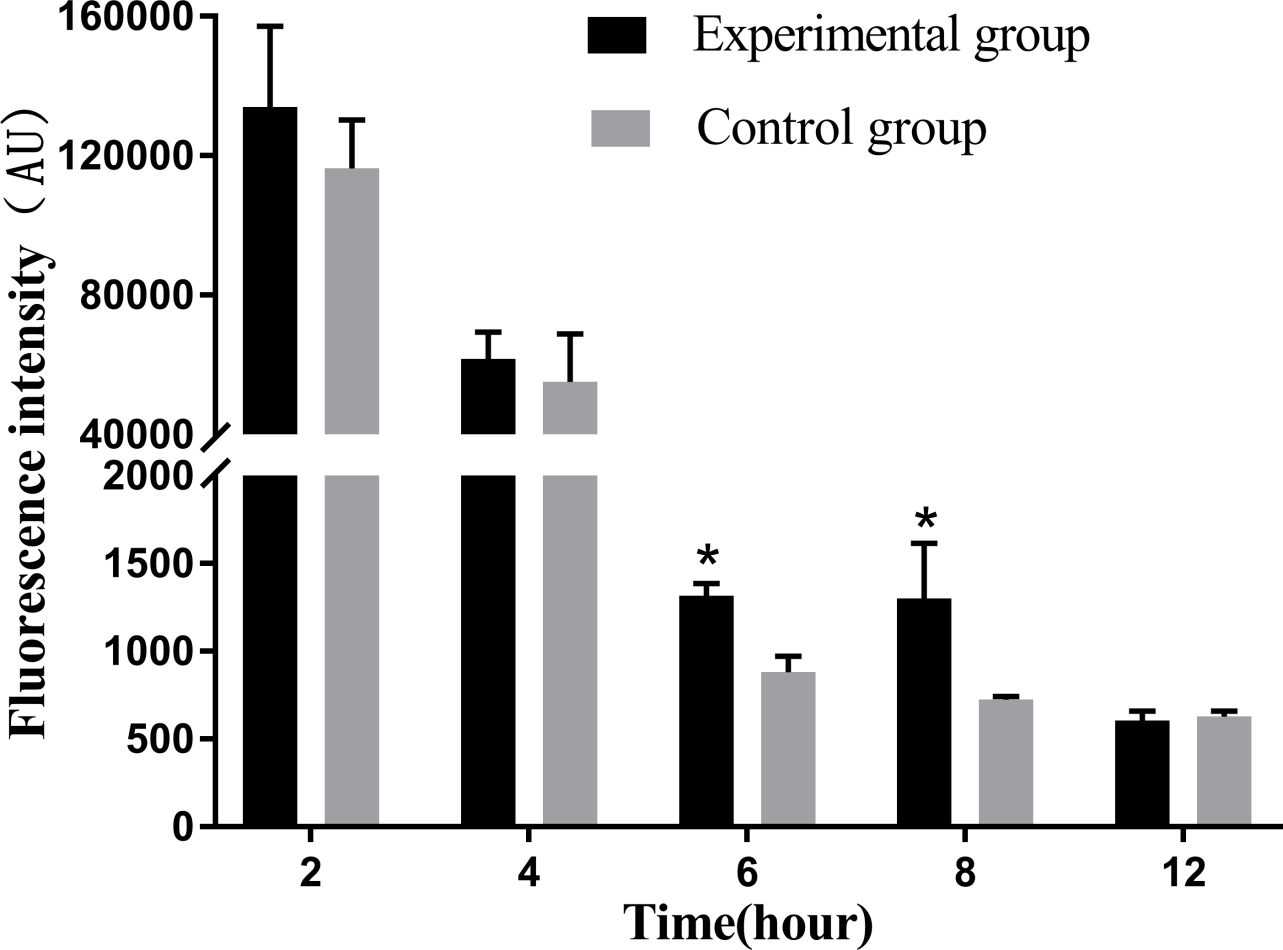
Release levels of reactive oxygen species at different time periods. The ROS release assay was repeated three times, and each experiment was performed in triplicate. Results are presented as mean ± SD. *P<0.05, compared with the control group.

Infection with *N. seriolae* induces respiratory burst activity and ROS production in macrophages. Detection of ROS production revealed that macrophages produced the strongest ROS at 2 hpi, which significantly decreased with time from 4 to 12 hpi compared to the previous stage. In particular, at 6–8 hpi, ROS production decreased significantly compared with that in the control group (*P* < 0.05). At 12 hpi, ROS production decreased to its lowest level and there was no significant difference between the experimental and control groups (**Figure 6**).

**Figure 7:**
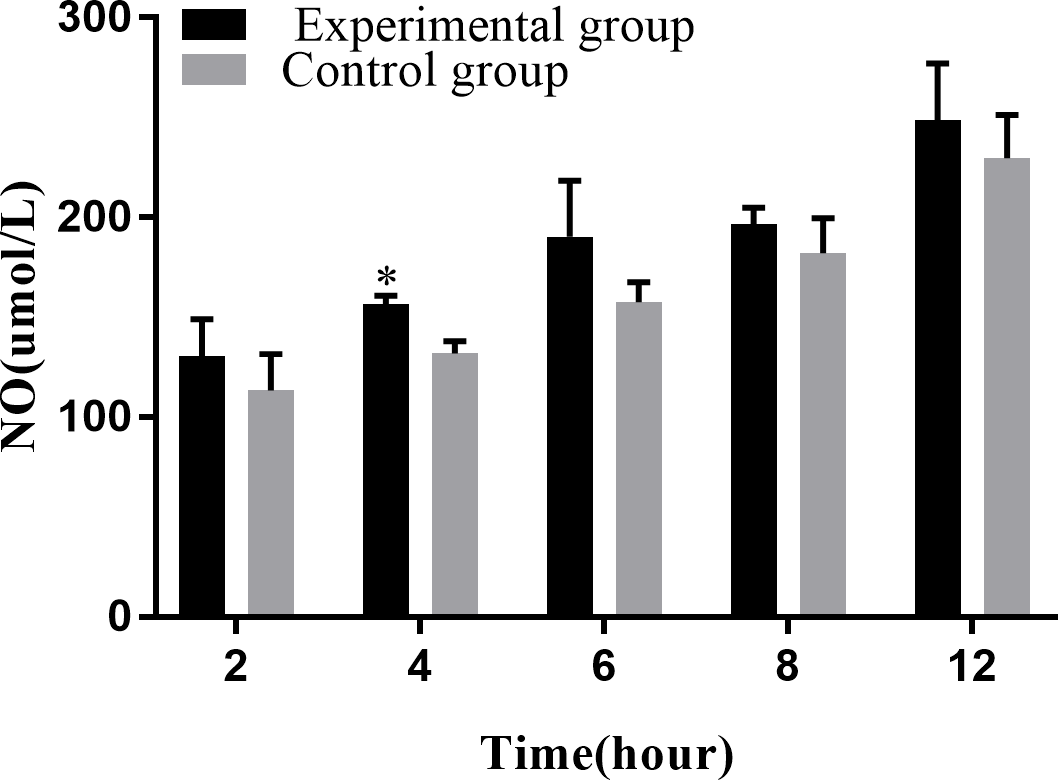
Levels of NO release at different time periods. The NO release assay was repeated thrice, and each experiment was performed in triplicate. Results are presented as mean ± SD. *P<0.05, compared with the control group.

NO plays an important role in the immune response of macrophages to foreign pathogens. Consistent with this, our results showed that the amount of NO released tended to increase with the infection time (**Figure 7**). At 4 hpi, the experimental group showed a significant increase compared with the control group during the same time period (*P* < 0.05).

### Apoptotic effect of *N. seriolae* on macrophage

**Figure 8:**
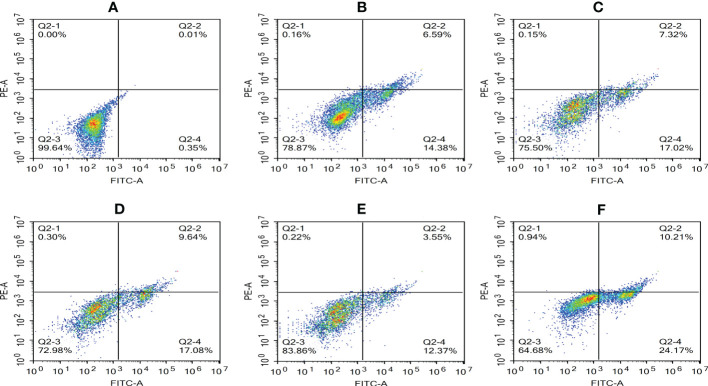
Proportion of apoptotic and necrotic cells at different time periods. **(A)** Uninfected cells: **(B)** 2 hpi, **(C)** 4 hpi, **(D)** 6 hpi, **(E)** 8 hpi, and **(F)** 12 hpi.

**Figure 9:**
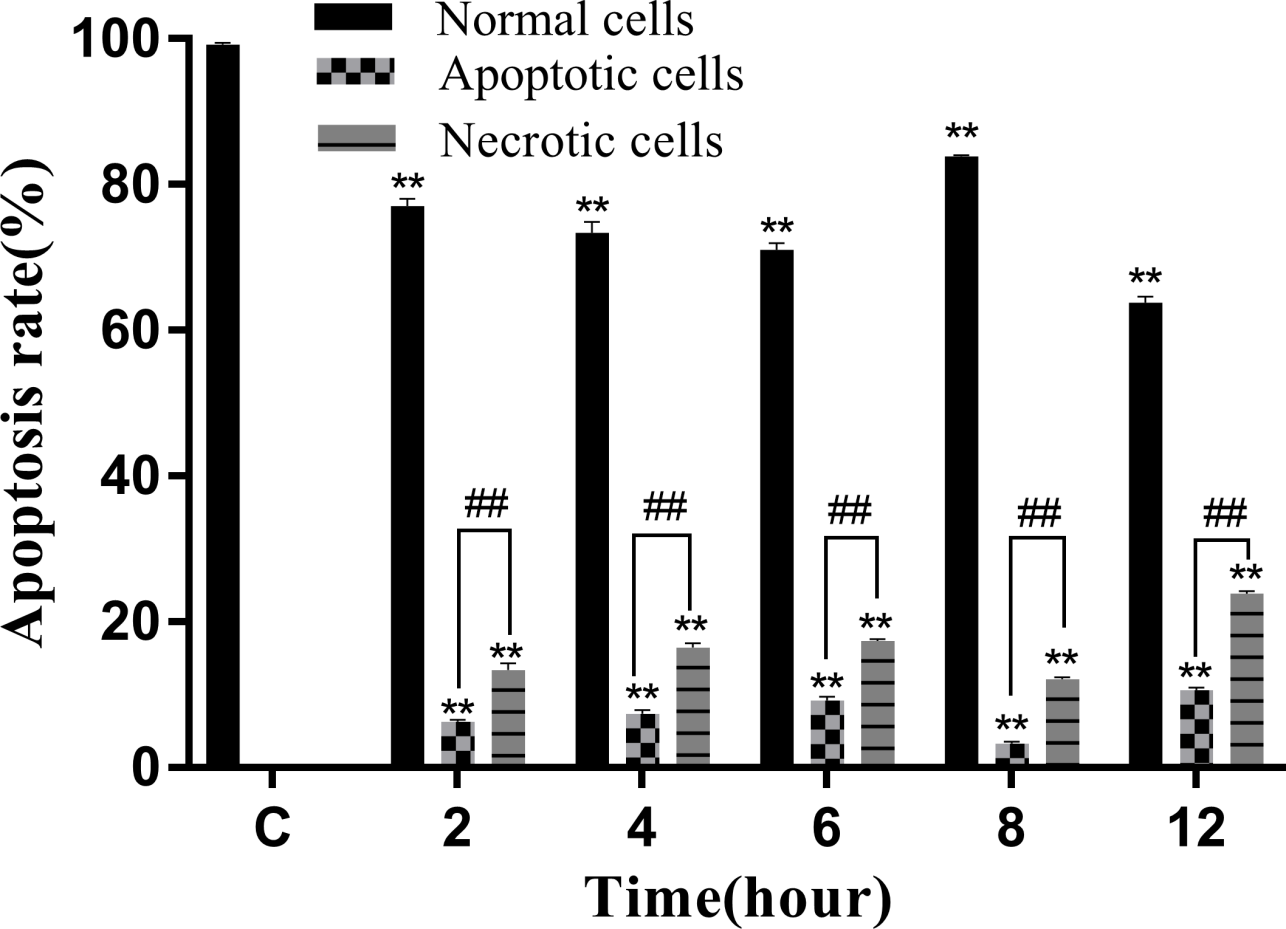
Apoptosis rate of RAW264.7 infected with *N. seriolae.* Data are presented as mean ± SD of three independent experiments. **P<0.01 as compared to the control group. ##*P* < 0.01indicates that the difference between apoptotic cells and necrotic cells at the same time point was highly significant.

The Annexin V-mCherry dual-fluorescence assay by flow cytometry was used to analyze early apoptotic and late apoptotic/necrotic cells. The findings displayed that the numbers of apoptotic and necrotic cells in the control group were within the normal range (**Figure 8A**). The proportion of apoptotic and necrotic cells increased from 6.75% and 14.38% to 9.94% and 17.08%, respectively, between 2 and 6 hpi (**Figures 8B–D**). At 8 hpi, the proportion of apoptotic and necrotic cells decreased to 3.77% and 12.37%, respectively (**Figure 8E**) but significantly increased to 11.15% and 24.17%, respectively, at 12 hpi (*P* < 0.05; **Figure 8F**). The results of the statistical analysis showed that the difference between apoptotic and necrotic cells was highly significant (*P*< 0.01) between each experimental group and the control group as well as between the experimental groups (**Figure 9**)

**Figure 10:**
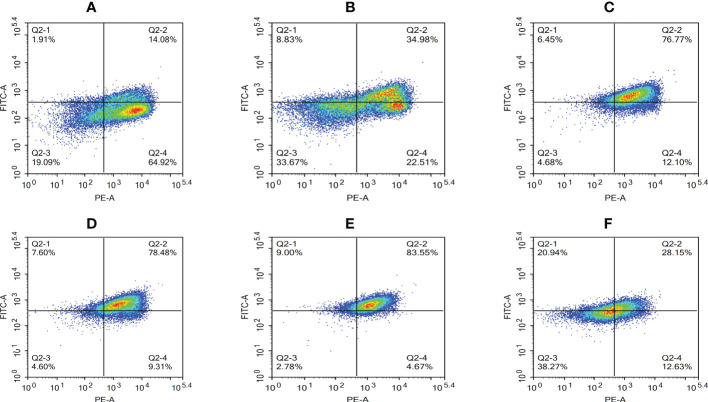
Proportion of cells with decreased mitochondrial membrane potential at different time periods. **(A)** Uninfected cells, **(B)** 2 hpi, **(C)** 4 hpi, **(D)** 6 hpi, **(E)** 8 hpi, and **(F)** 12 hpi.

**Figure 11:**
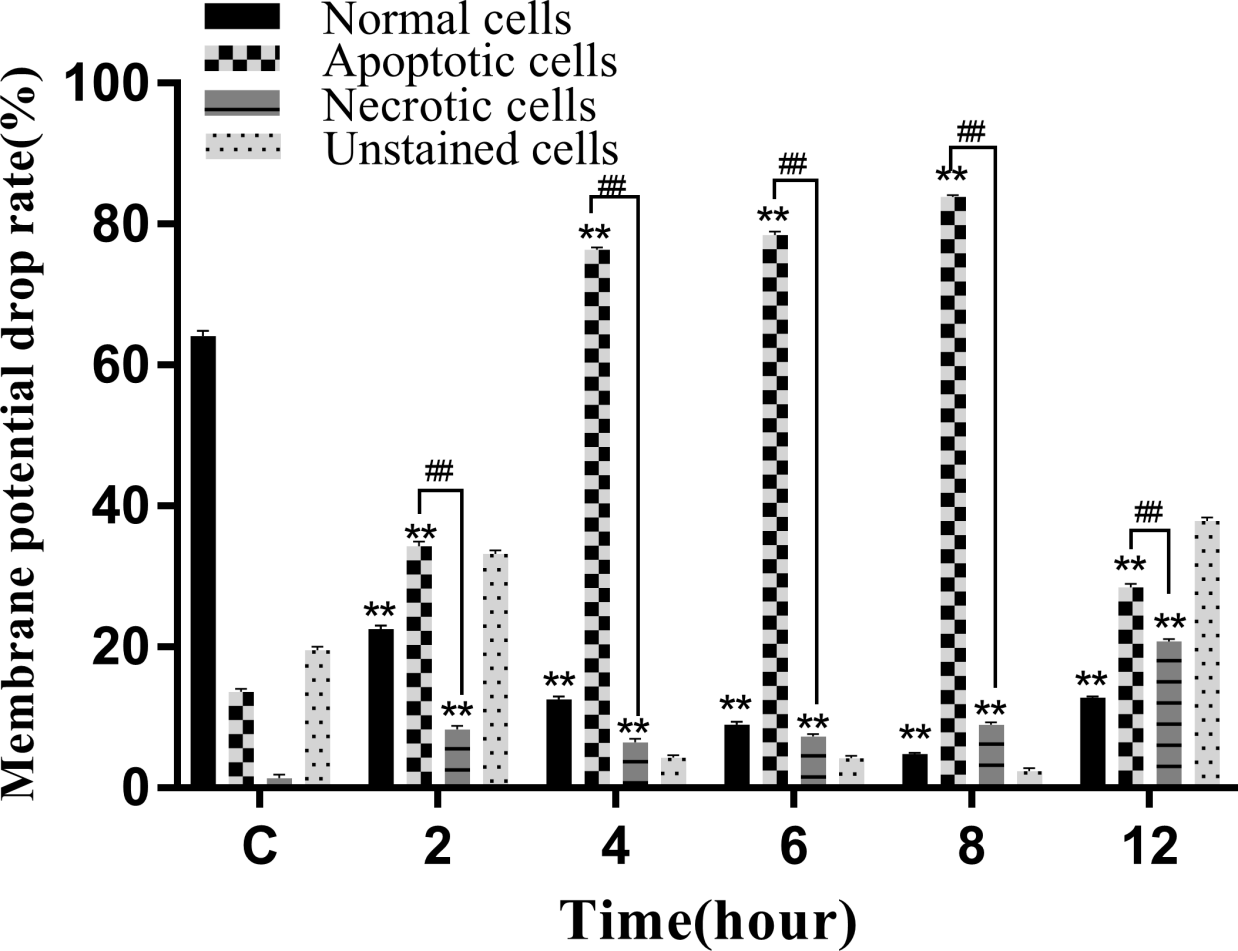
Membrane potential drop rate of RAW264.7 infected with *N. seriolae.* Data are presented as mean ± SD of three independent experiments. **P<0.01 as compared to the control group. ##*P* < 0.01indicates that the difference between apoptotic cells and necrotic cells at the same time point was highly significant.

A decrease in the mitochondrial membrane potential is an important sign of early apoptosis. We investigated changes in the mitochondrial membrane potential in infected macrophages using JC-1 staining, which selectively enters the mitochondria and changes color from red to green as a sign of decreased mitochondrial membrane potential. The results showed that the proportion of single-positive cells with red fluorescent probes in the control group was 64.92%, the proportion of double-positive cells was 14.08%, and the proportion of single-positive green fluorescent cells was 1.91%, which was within the range of normal cells (**Figure 10A**). After infection, the proportion of red fluorescent cells decreased and that of green fluorescent cells increased from 2 to 8 hpi, indicating that the infection induced mitochondrial disruption in macrophages (**Figures 10B–E**). At 12 hpi, the percentage of double-positive cells decreased significantly to 28.15%, indicating an increase in the number of necrotic cells (**Figure 10F**). The results of the statistical analysis showed that the difference between apoptotic and necrotic cells was highly significant (*P* < 0.01) between each experimental group and the control group as well as between the experimental groups (**Figure 11**).

**Figure 12:**
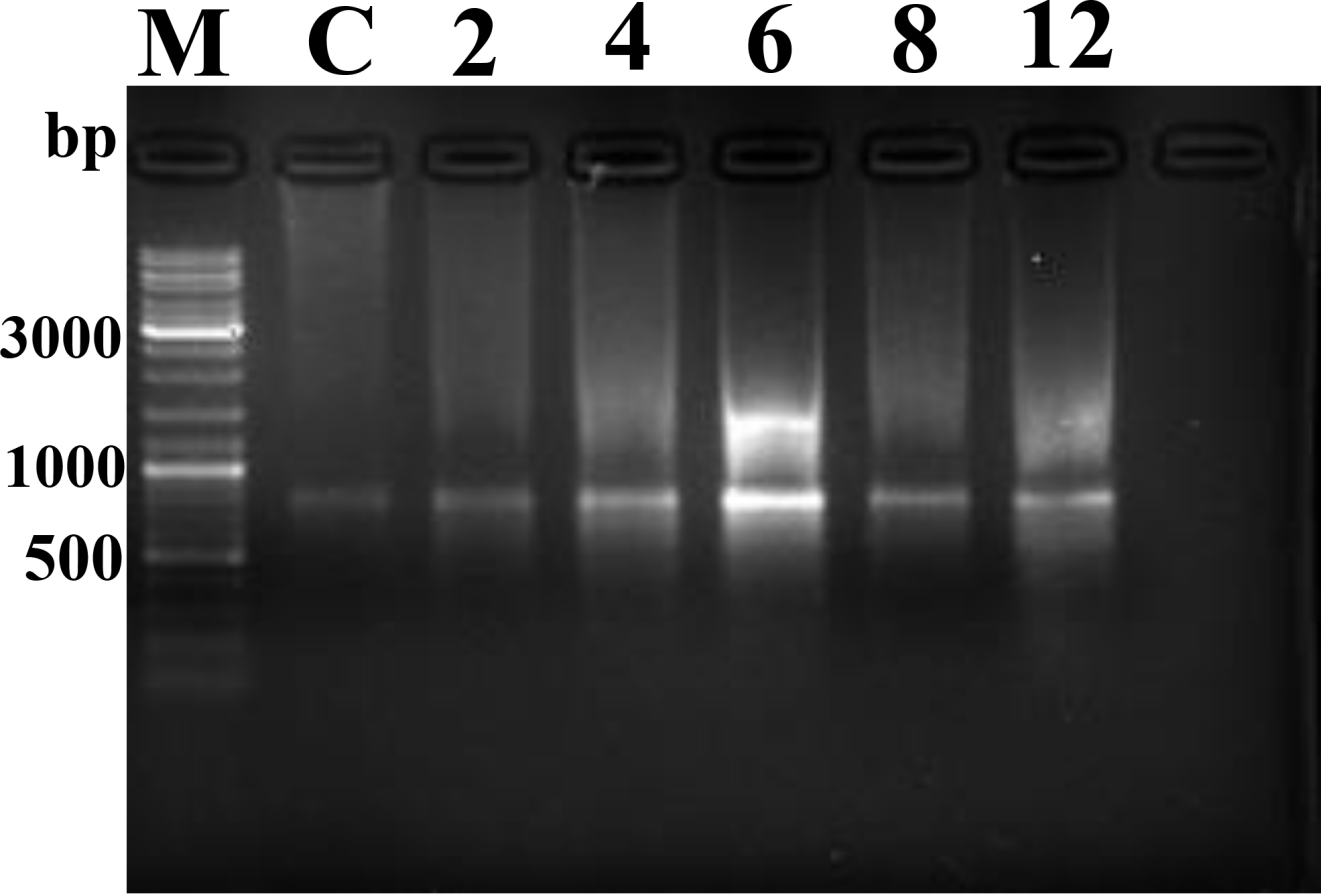
DNA Ladder assay results. M, Marker; C, control.

DNA fragmentation and orderly disintegration of cells and their organelles are among the final steps of the apoptotic process. DNA cleavage was assessed by DNA laddering testing. The results showed no obvious ladder-shaped bands at 2–4 hpi, whereas obvious ladder-shaped bands appeared at 6 hpi, which were more blurred and not easily observed after 8–12 hpi (**Figure 12**).

**Figure 13:**
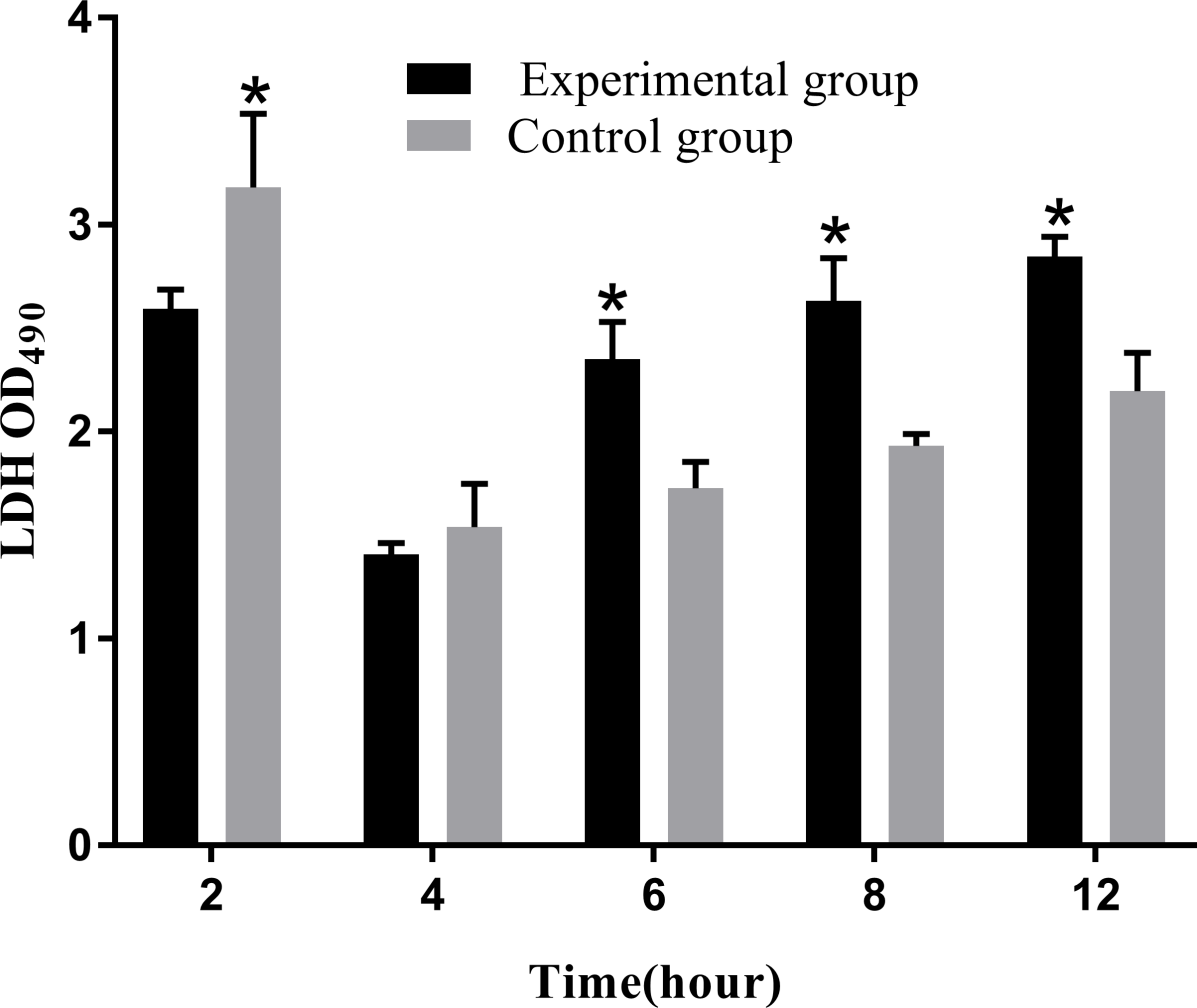
Levels of LDH release at different time periods. The LDH release assay was repeated three times, and each experiment was performed in triplicate. Results are presented as mean ± SD. *P<0.05, compared with the control group.

Apoptosis or necrosis can disrupt the cell membrane structure. The amount of LDH released directly reflected the integrity of the cell membrane. The results of LDH cytotoxicity detection showed that the release of LDH significantly increased from 6 to 12 hpi compared to that in the control group (*P*<0.05; **Figure 13**).

### Expression level of apoptosis factors and pro-inflammatory factors

**Figure 14:**
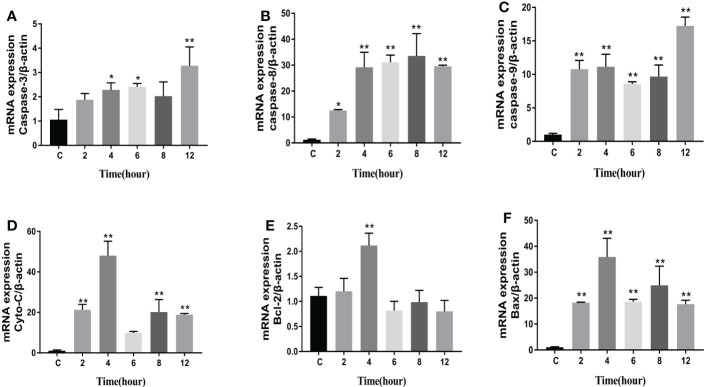
Expression level of apoptosis factors. **(A)**: Caspase-3; **(B)**: Caspase-8; **(C)**: Caspase-9; **(D)** Cyto-C, **(E)** Bcl-2, and **(F)** Bax. Data are presented as mean ± SD of three independent experiments. *P<0.05; **P<0.01, compared with the control group.

**Figure 15:**
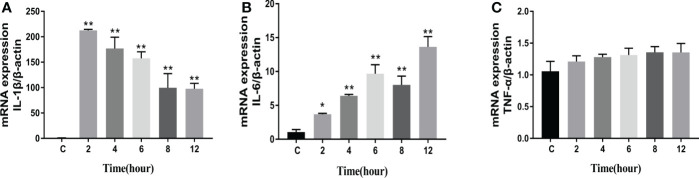
Expression level of pro-inflammatory factors. **(A)** IL-1β, **(B)** IL-6, and **(C)** TNF-α levels. Data are presented as mean ± SD of three independent experiments. *P<0.05; **P<0.01, compared with the control group.

The levels of apoptosis-related genes caspase-3, caspase-8, caspase-9, cyto-c, Bcl-2, Bax, and the pro-inflammatory factors IL-β, IL-6, and TNF-α in *N. seriolae*-infected macrophages were detected using RT-qPCR. The performance revealed that caspase-3 (**Figure 14A**), caspase-8 (**Figure 14B**), and caspase-9 (**Figure 14C**) were upregulated from 2 to 12 hpi compared with those in the control group (*P*<0.05). Cyto-c (**Figure 14D**), Bcl-2 (**Figure 14E**), and Bax (**Figure 14F**) were upregulated and showed the highest expression levels at 4 hpi, after which they were downregulated from 6 to 12 hpi. The relevant inflammatory factor (IL- β) in macrophages infected with *N. seriolae* was significantly downregulated (*P*<0.05; **Figure 15A**), whereas IL-6 was significantly upregulated from 2 to 12 hpi compared with that in the control group (*P*<0.05; **Figure 15B**). The mRNA expression level of TNF-α was not different during infection compared with that in the control group (**Figure 15C**). Collectively, these results suggested that macrophages infected with *N. seriolae* promoted the expression of apoptotic factors and inflammatory cytokines.

## Discussion

### Intracellular behavior of *N. seriolae*-infected RAW264.7 macrophages

The survival of host phagocytes is an essential virulence property of intracellular bacteria, such as *Brucella* spp. (Ahmed et al., 2016), *Edwardsiella tarda* (Qin et al., 2017), and *Mycobacterium tuberculosis* (Mishra et al., 2011), respectively. The fate of bacteria phagocytosed by macrophages depends on several factors, including the phagocytic state of macrophages and the specific virulence factors produced by the bacteria that allow them to survive the oxygen-dependent or-independent bactericidal products of macrophages (Beaman and Beaman, 1984). Macrophages can eliminate most bacteria from host tissues by the non-selective phagocytosis of exogenous microorganisms or by employing specific mechanisms during cellular immunity. In this study, microscopic observations and flow cytometry assays were performed to elucidate the intracellular behavior of *N. seriolae* in infected RAW264.7 macrophages.

After entering the macrophages, *N. seriolae* undergoes various intracellular changes. At 2 hpi, a small number of bacteria entered the cells, whose structures were apparently unchanged. After 4–6 h of infection, the number of bacteria entering the cells increased and they colocalized with the phagolysosomes. Inverted fluorescence microscopy and TEM observations confirmed that, at this time point, the macrophages were fused, larger, and contained more intracellular autophagic lysosomes and vacuoles. These results indicate that, once they enter macrophages, numerous *N. seriolae* can be phagocytosed by phagolysosomes, which are phagocyte defenses of macrophages, similar to the results obtained for *Brucella* (Ahmed et al., 2016), *E. tarda* (Qin et al., 2017), and *Vibrio harveyi* (Nguyen et al., 2018). Macrophages have an intrinsic ability to fight a variety of microbial pathogens and use their cytoskeletal dynamics to help maintain the integrity of natural immunity and host defense. When *P. aeruginosa* and *Streptococcus pneumoniae* are phagocytosed by macrophages, they may promote their own intracellular survival by influencing macrophage cytoskeletal actin dynamics, leading to pathogen elimination through autophagy (Jati et al., 2018). The results of this study showed that *N. seriolae* induced *in vitro* macrophage cytoskeleton movement and induced a migration response, leading to macrophage aggregation and fusion to form multinucleated macrophages. These multinucleated macrophages are more destructive to intracellular bacteria than individual macrophages (Beaman, 1977). This is consistent with the experimental results of *Nocardia asteroides* ATCC14579 infection of rabbit alveolar macrophages (Beaman, 1977). In contrast, neither the low-virulence strain of *N. asteroides* ATCC10905 nor the high-virulence strain of *N. asteroides* GUH-2 induces macrophage fusion (Beaman and Smathers, 1976; Beaman and Beaman, 1992; Bourgeois and Beaman, 1974). Confocal microscopy of *N. seriolae*-infected cells after 8–12 h of infection showed that the lysosomes and nuclei of the RAW264.7 cells appeared diffuse, which may be related to the growth and multiplication of *N. seriolae* in the cells during the late stages of infection. TEM revealed severe damage inside cells at 8–12 hpi, with a large expansion of phagocytic lysosomes and extensive nuclear lysis, which triggered apoptosis and partial inhibition of lysosomal fusion. These findings are consistent with the pathogenic mechanism of *N. asteroides* ATCC14579 (Beaman, 1977) and provide a basis for clarifying the pathogenic mechanisms of *N. seriolae* infections.

### Immune response to RAW264.7 macrophages infected with *N. seriolae*

When intracellular parasitic bacteria infect macrophages, they induce a series of immune responses, including the release of small inorganic molecules that kill foreign invaders. As important secondary messengers, ROS not only participates in the immune response of cells but also plays a major role in tumorigenesis and development (Rong et al., 2019). The strength of the bactericidal function of macrophages is mainly reflected in the amount of ROS released (Hafner et al., 2017), because ROS can regulate the polarization process of macrophages, including M1 and M2 types. M1 macrophages are usually induced by pathogen-associated molecular patterns (PAMP) and Th1-type cytokines such as interferon γ and tumor necrosis factor α. These activated macrophages produce pro-inflammatory cytokines and chemokines that contribute to the host defense against intracellular pathogens and tissue damage. Conversely, M2 macrophages with anti-inflammatory effects can be induced by a range of mediators, such as IL-4, IL-10, IL-13, and transforming growth factor β, which are involved in tissue repair, remodeling, and angiogenesis. It was shown that M1 macrophages are more susceptible to inflammation-associated necrotic cell death than M2 macrophages, which may be associated with the upregulation of necrotic signaling molecules such as RIPK3, MLKL and ZBP1 (Hao et al., 2021).

However, the generation of a large amount of ROS by macrophages can damage the macrophages themselves *via* processes such as the induction of somatic mutations and tumor transformation, and can even directly cause oxidative damage to DNA, leading to structural changes in DNA and subsequent apoptosis or necrosis (Guidarelli et al., 2017). In this study, the level of ROS release reached its highest value at 2 hpi, and ROS levels continued to decrease with increasing infection time, further confirming that *N. seriolae* achieves intracellular survival in macrophages by inhibiting ROS release.

NO, another small inorganic molecule, plays an important role in intercellular information transfer and immune regulation (Esquivel-Solís et al., 2013). NO production determines its specific role and is similar to the regulatory mechanism of ROS, with high concentrations exerting bactericidal effects. In this study, the levels of NO released continued to increase with increasing infection time, which is consistent with *Mycobacterium bovis* infection-induced NO production by macrophages (Luo et al., 2019). Infected macrophages produce large amounts of NO through a peroxidation mechanism to kill non-invasive or intracellularly colonized *N. seriolae*, whereas small amounts of NO act as messenger molecules to “wake up” macrophages to the immune defense phase and increase their ability to kill bacteria. It has been found that the main factor of bovine macrophage defense against *Mycobacterium avium* binding is NO production rather than apoptosis (Esquivel-Solís et al., 2013), further suggesting that NO production by macrophages has a positive effect on controlling *N. seriolae*.

Upon infecting RAW264.7, *N. seriolae* reaches the interior of macrophages through adhesion and invasion, forcing them to release relevant cytokines and produce an inflammatory response to kill exogenous pathogenic bacteria. However, the amount of inflammatory factors produced has different effects on cells: low production can kill intracellular bacteria, whereas high production can cause significant damage to cells. IL-1β is an important pro-inflammatory factor that plays a major role in cellular anti-infection processes. However, persistent production of IL-1β also leads to cellular damage (Dinarello, 2009; Sahoo et al., 2011). In this study, we found that the initial expression of IL-1β was extremely high at 2 hpi, which indirectly indicated that the infected cells produced an inflammatory response. During infection, the expression of IL-1β begins to decrease gradually, indicating that *N. seriolae* may inhibit the inflammatory response for survival by downregulating the expression of IL-1β, which is similar to that observed in *Listeria* (Meixenberger et al., 2010), *Salmonella* (Pie et al., 1996), and *M. tuberculosis* (Behar et al., 2011). IL-6 and TNF-α are the two most common cytokines. A previous study found that cell wall-associated lipids of *N. brasiliensis* promoted IL-6 production by macrophages and inhibited TNF-α production by macrophages during infection (Trevino-Villarreal et al., 2012). In our study, both IL-6 and TNF-α expression were upregulated, similar to that observed after *N. brasiliensis* infection (Trevino-Villarreal et al., 2012). These results also implied that in the initial infection stage, *N. seriolae* induced a macrophage inflammatory response, which was subsequently inhibited by the bacteria for its survival.

### Apoptotic effect of *N. seriolae* infection on RAW264.7 cells

Apoptosis is a genetically regulated programmed cell death (PCD) pathway. When a cell is infected with an exogenous intracellular bacterium, normal macrophages phagocytose and maintain a healthy state (Savill et al., 2002). Moreover, apoptosis is a non-hemolytic cell death, in which the dying cells are phagocytosed when the cell membrane is ruptured before cell rupture. Therefore, this process is usually immune and has little effect on the neighboring cells. Cell necrosis is a programmed and regulated form of cell death that causes cell membrane rupture and triggers inflammation through the release of damage-associated molecular patterns (DAMP), which include immunostimulatory intracellular components such as high-mobility histone B1, IL-1 family cytokines, nucleic acids and S100 proteins (Kaczmarek et al., 2013; Pasparakis and Vandenabeele, 2015).It is important to note that apoptosis is triggered by either an exogenous (cell surface receptor) or endogenous (mitochondrial) apoptotic signaling pathway, which leads to the activation of caspase-9 and caspase-8, respectively (Wen et al., 2012). Both pathways converge by activating caspases 3, 6, and 7, which in turn activate substrates that mediate cellular morphological changes. In contrast, cell necrosis does not involve caspase-dependent pathways but includes major molecules such as mixed-spectrum kinase domain-like proteins (MLKL) and receptor-interacting serine/threonine kinases (e.g., RIP1 and RIP3.) RIP1 kinases are also been found to be involved in apoptosis, whereas RIP3 and MLKL are only associated with cell necrosis. One of the major mechanisms of cellular necrosis is the formation of a necroptotic complex between RIP1-RIP3-MLKL, phosphorylation of MLKL proteins ultimately drives membrane pore formation, and cells undergo osmotic pressure changes with the release of environmental and danger-associated molecular patterns (DAMP), which further increases inflammation (Kaczmarek et al., 2013). In this study, we found that the mitochondrial membrane potential decreased and pre-apoptotic genes (Bcl-2, Bax, cyto-c, caspase-8, and caspase-9) were significantly upregulated. This implies that the infected macrophages promoted apoptosis to eliminate intracellular bacteria and prevent proliferation and continuous infection with *N. seriolae.* These results are consistent with those reported for *M. tuberculosis* (Behar et al., 2011), where avirulent mutants of *M. tuberculosis* induced more apoptosis than the virulent strain, indicating that virulent *M. tuberculosis* can inhibit apoptosis in macrophages (Behar et al., 2011). At the middle time points of infection with *N. seriolae*, the number of apoptotic cells decreased, probably because the apoptotic cells sent a “eat me” signal to the uninfected macrophages, inducing increased clearance of apoptotic cells; such a process is called “efferocytosis” (Jäger et al., 2021). Some intracellular bacteria, such as *M. tuberculosis*, have evolved to inhibit apoptosis. *M. tuberculosis* suppresses apoptosis by inhibiting ROS accumulation and upregulating anti-apoptotic gene transcription by activating the NF-κB signaling pathway (Jurcic Smith and Lee, 2016). We also observed a decrease in ROS and NO production, similar to that observed after *M. tuberculosis* infection, implying that *N. seriolae* might inhibit apoptosis for survival inside macrophages. In the late stages of infection, a large number of cells were infected with *N. seriolae*, and the numbers of both apoptotic and necrotic cells continued to increase. In this case, apoptosis often leads to the subsidence of inflammation caused by *N. seriolae*; however, cell necrosis can cause the persistence of inflammation, thus prolonging the time the cells are infected. Thus, even after cell death, they promote the inflammatory process, allowing host cells to enhance the immune process. (Kundu et al., 2022) Furthermore, the increased amount of LDH suggests that the cells ruptured and disintegrated. TEM observations further confirmed chromatin condensation, fragmentation, and formation of apoptotic vesicles.

## Conclusion

In conclusion, we characterized the intracellular behavior of *N. seriolae* in the infected RAW264.7 macrophage cell line model. The results showed that *N. seriolae* was capable of invading and surviving inside the macrophages. We further demonstrated that *N. seriolae* induced apoptosis in macrophages early after infection and inhibited apoptosis for intracellular survival in the middle stage of infection. This study provides the first comprehensive insight into the intracellular behavior of *N. seriolae* and its apoptotic effects on macrophages. These findings may be important for understanding the pathogenicity of nocardiosis in fishes.

## Data availability statement

The original contributions of this study are included in the article and **Supplementary Material**. Further inquiries can be directed to the corresponding author.

## Author contributions

WL, YT, and AT analyzed the data and performed the experiments. FW and YL offered the experimental materials. OC helped with microscopic observations. WL and YT wrote the manuscript. FZ and ZH designed the study and conceived of the project. All the authors contributed to the article and approved the submitted manuscript.

## Supplementary material

Supplementary Table 1 Primer sequences for apoptosis and inflammatory factors.
