# Supplementary material for: Intracellular behavior of Nocardia seriolae and its apoptotic effect on RAW264.7 macrophages
Source: Front Cell Infect Microbiol. 2023 Feb 28;13:1138422. doi: 10.3389/fcimb.2023.1138422 (PMC10011490; doi:10.3389/fcimb.2023.1138422)
Supplement: Supplementary Table 1 — Primer sequences for apoptosis and inflammatory factors. [file Table_1.docx]

Supplementary Material

**Table S1 Primer sequences for apoptosis and inflammatory factors**

| **Gene target** | **Primers** | **Sequence (5’-3’)** | **Annealing temperature (℃)** | **References** |
| --- | --- | --- | --- | --- |
| TTBax | Bax-F  Bax-R | CAGGATGCGTCCACCAAGAA  CAAAGTAGAAGAGGGCAACCAC | 60℃ | Wang et al., 2022 |
| Bcl-2 | Bcl-2-F  Bcl-2-R | CTACGAGTGGGATGCTGGAGA  CAGGCTGGAAGGAGAAGATGC | 60℃ |  |
| Caspase-3 | Caspase-3-F  Caspase-3-R | GGCTGACTTCCTGTATGCTTACTCTAC  ACTCGAATTCCGTTGCCACCTTC | 62℃ |  |
| Caspase-8 | Caspase-8-F  Caspase-8-R | ACCAAATGAAGAACAAACCTCG  CTTCATTTTTCGGAGTTGGGTT | 60℃ |  |
| Caspase-9 | Caspase-9-F  Caspase-9-R | CGCCAAAATTGAAATTCAGACG  CGACAGGCCTGGATGATAAATA | 60℃ |  |
| Cyto-C | Cyto-C-F  Cyto-C-R | GCAGGGTGCTAACTCAGTCC  CACTTAGGATCACCCCCAGC | 58℃ |  |
| IL-6 | IL-6-F  IL-6-R | CGGAGAGGAGACTTCACAGAG  ATTTCCACGATTTCCCAGAG | 60℃ |  |
| IL-1β | IL-1β-F  IL-1β-R | GCACTACAGGCTCCGAGATGAAC  TTGTCGTTGCTTGGTTCTCCTTGT | 62℃ |  |
| TNF-α | TNF-α-F  TNF-α-R | TACTGAACTTCGGGGTGATTGGTCC  CAGCCTTGTCCCTTGAAGAGAAC | 62℃ |  |
| GAPDH | GAPDH-F  GAPDH-R | CGTGCCTGGAGAAACCTG  AGAGTGGGAGTTGCTGTTGAAGTCG | 62℃ |  |

**References:**

Wang, F. F., Zhao, P. Y., He, X. J., Jiang, K., Wang, T. S., and Xiao, J. W., et al. (2022). *Fusobacterium necrophorum* promotes apoptosis and inflammatory cytokine production through the activation of NF-κb and death receptor signaling pathways. *Frontiers in Cellular and Infection Microbiology.* 12:827750. doi:10.3389/fcimb.2022.827750
